# Minimally Invasive Beaded Electrosurgical Dissectors, Basic Science, and Pilot Studies

**DOI:** 10.1093/asjof/ojae034

**Published:** 2024-05-06

**Authors:** Taiyo C Weber, Mark Jewell, Carl I Schulman, Jefferson Morgan, Alison M Lee, Alicia K Olivier, Elizabeth A Swanson

## Abstract

**Background:**

Minimally invasive beaded electrosurgical dissectors (“BEED devices”) provide simultaneous sharp dissection, blunt dissection, and electrosurgical coagulation while performing 100 cm^2^ porcine tissue plane dissections in 0.8 to 3 min with minimal bleeding and no perforations.

**Objectives:**

The aim of the study was to report the basic science and potential clinical applications and to video document the speed and quality of planar dissections in in vivo and ex vivo porcine models with thermal damage quantified by thermal and histopathologic measurements. Additionally, in vivo porcine specimens were followed for 90 days to show whether adverse events occurred on a gross or macroscopic basis, as evidenced by photography, videography, physical examination, and dual ultrasonography.

**Methods:**

Ex vivo porcine models were subjected to 20, 30, and 50 W in single-stroke passages with BEED dissectors (granted FDA 510(k) clearance (K233002)) with multichannel thermocouple, 3 s delay recordation combined with matching hematoxylin and eosin (H&E) histopathology. In vivo porcine models were subjected to eight 10 × 10 cm dissections in each of 2 subjects at 20, 30, and 50 W and evaluated periodically until 90 days, wherein histopathology for H&E, collagen, and elastin was taken plus standard and Doppler ultrasounds prior to euthanasia.

**Results:**

Five to 8 mm width dissectors were passed at 1 to 2 cm/s in ex vivo models (1-10 cm/s in vivo models) with an average temperature rise of 5°C at 50 W. Clinically evidenced seromas occurred in the undressed, unprotected wounds, and resolved well prior to 90 days, as documented by ultrasounds and histopathology.

**Conclusions:**

In vivo and ex vivo models demonstrated thermal values that were below levels known to damage subcutaneous adipose tissue or skin. Tissue histopathology confirmed healing parameters while Doppler ultrasound demonstrated normal blood flow in posttreatment tissues.

**Level of Evidence: 4:**

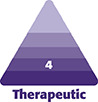

It has been just over a century since the invention of electrosurgery by William Bovie. Currently, electrosurgery is used in upwards of 80% of surgical procedures.^1^ Electrosurgery is a powerful and effective surgical tool. Since its conception, improvements have been continuously developed and studied.^2-16^ To date, there has not been an electrosurgical device that could be introduced through a minimally invasive entrance port into the subcutaneous tissue to dissect and create uniform, relatively bloodless tissue planes (exceeding 10 cm^2^) within 0.8 to 3 min depending upon tissue density.

Minimally invasive Beaded Enhanced Electrosurgical Dissectors (BEEDs) provide simultaneous sharp dissection, blunt dissection, and electrosurgical coagulation in a single surgical tool. BEED devices may dissect tissue planes with minimal bleeding and no gross planar perforations through entrance ports that are less than the diameter of a pencil. BEED devices are intended to function within or around soft tissues, but not within bone or cartilage. Benefits may include reduced operating times and modulated heat loading. Heat loading may be predictably controlled by the comparatively extreme rapidity of tip motion in a relatively static target tissue environment of gross tumescence. Furthermore, tissue modifying devices may be inserted into predissected planes formed by BEED devices to achieve other desired effects for tissue contraction or device implantation.

The objectives of the studies were to determine whether beaded surgical devices provided rapid plane dissections with an acceptable thermal effect, as evidenced by temperature and histopathologic measurements in in vivo and ex vivo models. Additionally, the in vivo porcine model was examined at 90 days postsurgery to determine whether adverse events occurred on a gross or macroscopic basis, as evidenced by photography (visible and infrared), videography, physical examination, and dual ultrasonography (standard images and Doppler).

## METHODS

### The BEED Device and Technique

**Figure 1.**
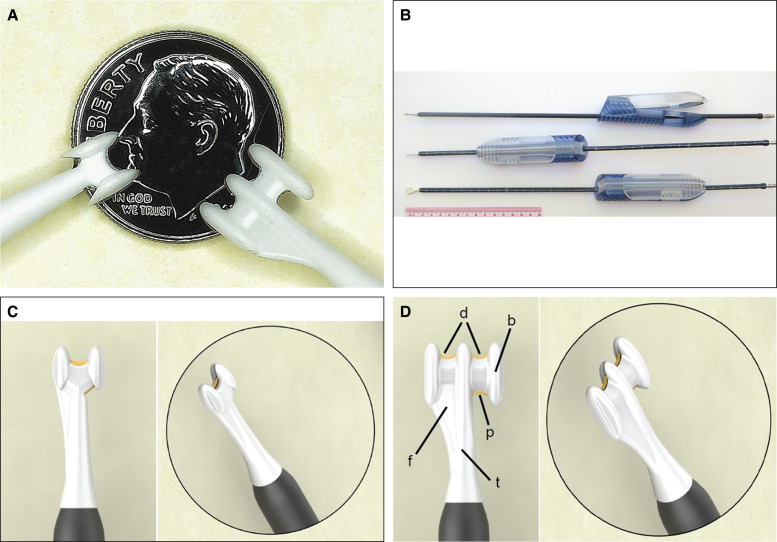
(A) Photographs of 2-BEED and 3-BEED models against 18 mm diameter US dime. (B) Ceramic tips atop 40 cm shafts with reversable and slidable snap handle. Top handle in unclamped position. (C, D) Computer-Aided Design (CAD) renderings of 2-BEED and 3-BEED devices show metallic conductive lysing segments in gold color detailing: distal lysing segments (d), proximal lysing segment (p) adjacent to bead (b) on ceramic tip (t). BEED, Beaded Enhanced Electrosurgical Dissectors.

As seen in Figure 1A, 2 initial device models have been developed: the 2-BEED-5mm (max-tip-width) model (“2-BEED”), and the 3-BEED-8mm (“3-BEED”). The devices utilize ceramic tips, wherein the first BEED number indicates the number of distal beads and/or “bead-like” structures present. Ceramic molding and 3D ceramic printing were used to manufacture the ceramic components. Nanoindentation testing indicates that BEED zirconia ceramic components tolerate approximately 1.38 × 1010 Pa (2 million psi).

Figure 1B illustrates the 2-BEED and 3-BEED ceramic tips fixed to a slightly flexible, 40 cm long Kynar (Arkema, France) plastic-sheathed shaft with reference marks at 1 cm intervals. The shaft allows for a multipurposed plastic snap handle to slide into any desired position, depending upon the distance from the entrance incision to the target tissue. The adjustable handle, with an internal wave-like shaft path, prevents shaft slippage to a force exceeding 90 N. The shaft allows for deliberate curving/bending of the device to place the tips at varying angles in target tissues that may be at greater distances from an entrance incision. The handle may be reversed depending on surgical site preference.

The metallic conductive lysing segments may be somewhat inconspicuous in photographs; however, the Computer-Aided Design (CAD) rendering in Figure 1D more clearly displays the exposed edges of 2 electroconductive stainless steel distal lysing segments (d) and 1 proximal lysing segment (p) adjacent to the rightmost bead (b) on the ceramic tip (t) of a 3-BEED model. Although the lysing segments may be beveled/sharpened to enhance both edge current concentration along with the lysing segment and current dispersion into tissues, the radiofrequency (RF) current lysing effect occurs sequestered between the bead tips, and at minute distances anterior to the lysing segments. The nonconductive bead-tip surfaces may stretch, spread, guide, and/or position (blunt dissection feature) the target tissue for lysing while sequestering the electrosurgical energy between the nonconductive bead tips.

In subcutaneous tissue dissections, the device may be operated somewhat similarly to a traditional liposuction cannula, in a spoke-wheel fashion with re-angled back-and-forth motions, originating in a zone adjacent to the entrance incision. Tumescent fluid infiltration (favoring Lactated Ringers Solution containing epinephrine) coupled with multipoint traction by an assistant is recommended. Multipoint traction is further achieved by the surgeon placing a skin hook in the entrance incision and pulling the hook toward the surgeon; this simple multitasking opens the entrance incision while contributing to overall multipoint traction. As in liposuction, palpation by the surgeon over the tip's path may be desirable, especially over curviform tissue surfaces. The forward or rearward force applied by the surgeon is necessary to bring the tissues that will be lysed into proximity with an energized lysing segment. The bead-tips and lysing segments allow for proportionately faster dissections with natural tissue density and planarity, playing a large role in controlling or restricting the instrument's path.

To create the plane of dissection, the tip is passed back-and-forth in a spoke-wheel fashion from the entrance incision to remove all fibrous attachments. Electrosurgical generator (ESG) energy levels within the range of 10 to 50 W with most being at 30 to 40 W. Once the area is undermined, a powerless “clean sweep” is performed to test for remaining fibrous attachments; if any resistance is encountered, the proximal bead is backstroked to encounter the resistant elements, and power is then engaged to lyse the specific element. The process is repeated until an unpowered dissector can be passed through the entire target area without encountering any resistance.

The presence of a proximal lysing segment and a tissue deflector/fender (Figure 1D) allows for the benefit of fewer beads and thus less device width, minimizing the entrance wound and facilitating a unidirectional-vectored coordinated withdrawal force. The proximal assembly allows rearward lysing, while the tissue deflector/fender prevents the contralateral bead from catching tissue on proximally vectored (withdrawal) strokes. Therefore, the option of combined bi-directional, 2-way (forward/distal/anterograde and reverse/proximal/retrograde) motion may improve dissection efficiency. Allowing retrograde dissection may favor additional dissection angles or patterns when various force vectors are placed on the target tissues and/or target tissue planes. The device can be inverted (flipped over) 180°, depending on the surgeon's handedness or other preferences.

### Beaded Enhanced Electrosurgical Dissectors (BEED) Device Evaluation Studies

BEED device evaluation studies included testing in ballistic gelatin models, fresh ex vivo porcine tissue models, and in vivo porcine models with a 90 day follow-up was undertaken between January 2020 and September 2023.

### Ballistic Gelatin-Simulated Tissue Model

A superconcentrated ballistic gelatin structure that contained pseudo-tendrils (simulating collagen fibers) was prepared with sufficient clarity to facilitate video photographic documentation. The passage of the BEED devices was captured by video to demonstrate dissection speed and discharge of the RF energy.

### Ex Vivo Porcine Model

Fresh ex vivo porcine external abdominal tissue, including epidermis, dermis, subcutaneous fat, fascia, and muscle, was obtained from an abattoir and secured to plastic-coated plywood with metallic “trim” screws. A 2-BEED device was powered by a Bovie Icon GP ESG (Apyx Medical, Clearwater, FL) set on “Blend” at 20/30/50 W power. The BEED device was evaluated in both porcine subcutaneous adipose tissue (SAT) and porcine muscle to determine tissue response to the RF energy that was delivered by the device. No tumescence was used because, on pilot testing, almost all of the tumescent fluid immediately leaked from the subcutaneous edges of the specimen and was determined to be a concern for an electrical hazard to personnel. An infrared video camera (FLIR ONE Edge Pro by Teledyne-FLIR LLC, Wilsonville, OR) was mounted approximately 18 inches above the tissue specimen; compressed foam sheets leaving exposed only the tissue being treated were used to minimize outside thermal signatures that could confound the software tracking maximum temperature “hot spots.” Three probes connected to a multichannel thermocouple (PerfectPrime TC0520) measured and logged every 5 s the temperature of the ambient environment (at body temperature), the center of the tissue strip, and the maximum temperature found in the path/tunnel within 1 to 3 s after BEED device passage.

The application of tissue marking dye-blue (MOPEC, Madison Heights, MI) into the dissection paths/tunnels, in addition to a multifilament thread, was utilized to mark the track of the device. Tissue samples encompassing the length of a tissue path/tunnel were excised and immediately placed in 10% neutral buffered formalin. Two cross sections were taken along the length of each device path approximately 1 to 2 mm apart, grossed, embedded in paraffin, sectioned, and stained with hematoxylin and eosin (H&E). Histological evaluation for the ex vivo model was performed by a board-certified pathologist (J.M.) who rated the depth of the thermal effect using an Olympus micrometer (Evident Corporation, Japan) and calibration slide. The depth of tissue thermal effect was measured upon noting any degree of tissue pallor, eosinophilia, or loss of cellular detail.

### In Vivo Porcine Model: subcutaneous adipose tissue (SAT)

The BEED device was evaluated in an in vivo porcine tissue model with a 90 day follow-up period. All procedures used in this study were approved by the Mississippi State University Institutional Animal Care and Use Committee. Two healthy, 6-month-old, female, purpose-bred Yorkshire-Landrace crossed pigs were cared for in accordance with the Guide for the Care and Use in Laboratory Animals. Pigs were housed together preoperatively, individually for 2 weeks postoperatively to heal, and then together again for the remainder of the study. The room was maintained at a temperature range of 61°F to 81°F with a 12 h:12 h light–dark cycle. They were fed Ware Milling pig grower feed twice daily. The pigs were administered ceftiofur perioperatively (5 mg/kg IM q24 h × 3 days) starting 1 day prior to the surgery. For postoperative pain management, the pigs were administered carprofen (3 mg/kg SQ once) at the time of surgery, followed by carprofen (3 mg/kg PO q24h × 7 days) starting the day after surgery.

For the initial surgery, the pigs were premedicated with an intramuscular injection of telazol, ketamine, xylazine, and morphine. Once under deep sedation, each pig was intubated, and anesthesia was maintained on a mixture of isoflurane and oxygen. IV Ringer's lactate was administered, and depth of anesthesia, temperature, heart rate, respiratory rate, SpO_2_, ET CO_2_, and blood pressure were continuously monitored.

Pigs in dorsal recumbency were hair clipped and prepped with chlorhexidine and alcohol. Each was tattooed with eight 10 × 10 cm grids (6 abdominal, 2 medial thigh) for a sterile operating room, and each was given 1 of 16 labels: A1 to A8 and B1 to B8. Local anesthesia of 1 mL of 1% lidocaine and epinephrine was injected in a medial grid corner, followed by a 5 mm, 45° beveled #15 blade stab incision, followed by subcutaneous tumescent fluid instillation (500 mg lidocaine, 1 mg epinephrine per 1 L lactated Ringer's solution) under each grid in a fanning/spoke-wheel fashion through a 3 mm spatulated cannula or a spinal needle at 100 mL per 100 cm^2^. A small, <2 × 2 cm subcutaneous pocket was created using blunt Metzenbaum scissors.

Ten minutes were allowed for the epinephrine to take effect. The tip of the dissecting device was then introduced into the 2 × 2 cm pocket. Subcutaneous BEED dissections were carried out beneath each grid. A skin hook was firmly placed in the lower dermis of the entrance incision and pulled toward the surgeon, because the BEED dissector was pushed forward and pulled rearward to create and link dissection “spoke” paths. This was accompanied by continued proximal vectored tension through skin hook (and other traction vectors, as previously discussed) for the remainder of the spoke-wheel dissection, creating a plane in the SAT, as previously described. Following spoke-wheel dissection pattern completion, an attachment checking “clean-sweep” passage is performed with slight force being applied primarily in the retrograde vector with a component favoring the outer bead tip adjacent to the proximal lysing segment. Remaining tumescent fluids were milked gently through a 2 cm diameter rolling pin and incisions sutured using single buried 3-0 poliglecaprone. Ventral photographs were taken immediately preoperatively and postoperatively (immediately/7/30/60/90 days) while sedated with IM ketamine and xylazine. A hand-held FLIR ONE Edge Pro IR camera monitored skin surface temperature during device passage. The FLIR field of view at a practical distance from the surgical field included an additional area extending 20 cm out from the perimeter of the grids.

At 90 days postoperative, pigs were heavily sedated with IM telazol, xylazine, and ketamine. After obtaining 3 month ventral photographs, each grid was evaluated by ultrasound followed by IV pentobarbital overdose euthanasia (1 mL/10 lb). A 2.5 cm × 2.5 cm square full-thickness deep section of the tissue was harvested from grid centers for histopathological evaluation along with 2 additional, untreated-zone control specimens.

### Ultrasound Imaging

To evaluate tissue response to the BEED dissection after 90 days, the tissues deep in each grid (labeled A1-A8 and B1-B8) were examined using a 3 to 10 MHz microconvex transducer and a 12 to 20 MHz linear array transducer with a GE Logiq S8 Ultrasound machine (GE, Boston, MA). In addition, 3 control locations (1 lateral to the cranial treatment regions, 1 between treatment regions, and 1 on midline) were interrogated. The regions were also interrogated with color Doppler and power Doppler to evaluate for changes in blood flow.

### Histopathological Evaluation

Eight skin samples (“grids”) were evaluated from each pig (denoted A1-A8 and B1-B8). Three control samples were evaluated per pig. For each skin sample, the tissue was sectioned in half to obtain the midline section and transected in quarters to obtain the lateral sections. All sections were evaluated with H&E. Midline sections were also assessed with trichrome to evaluate fibrosis and Van Gieson to evaluate elastin.

## RESULTS

### Ballistic Gelatin-Simulated Tissue Model Results

**Figure 2.**
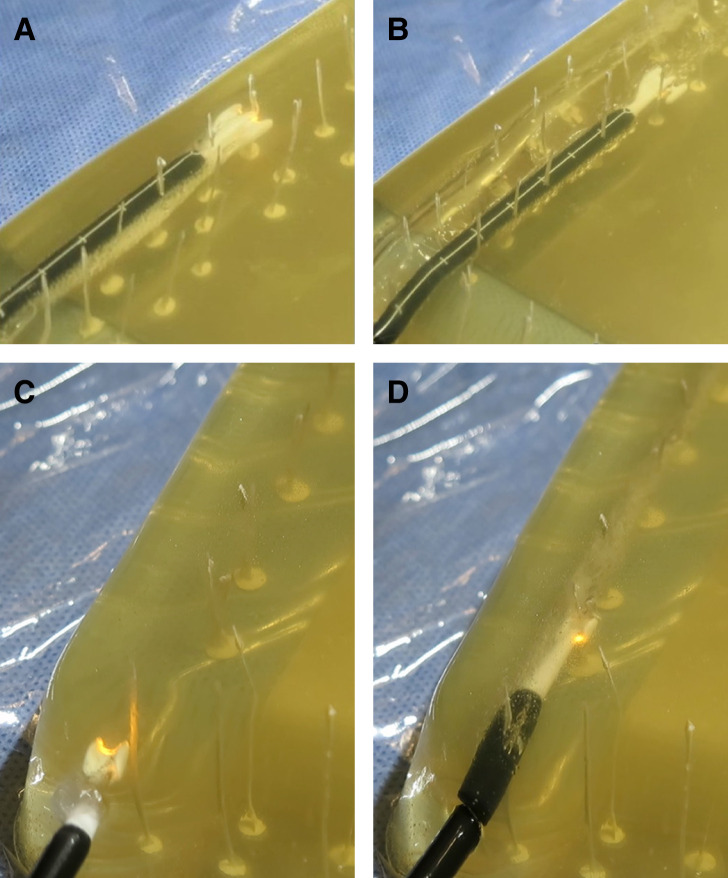
(A) 3-BEED model in ballistic gelatin lysing row of pseudo-tendrils (white) on forward motion. (B) 3-BEED model lysing in rearward motion with remnant bubble paths. (C) 2-BEED model in forward motion with arcing visible. (D) 2-BEED model in rearward motion lysing pseudo-tendrils in proximal segment. BEED, Beaded Enhanced Electrosurgical Dissectors.

In the translucent, superconcentrated ballistic gelatin with 2 rows of white pseudo-tendrils, the BEED devices were passed to and fro at approximately 1 cm/s when compared with the in vivo porcine model in which the BEED device's passage speed ranged from 1 to 10 cm/s depending upon target tissue type, density, vascularity, and location (Figure 2). The shaft's embossed 1 and 10 cm increment markers may visually aid the surgeon in estimating the speed of the tip. Electrical discharge arcs can be seen at the distal lysing electrodes (Video).

### Ex Vivo Porcine Model Results

Electrical discharge RF interference prevented the use of real-time common thermocouple data collection during electrosurgical dissection. The delay time of 1 to 3 s involved inserting the thermocouple into the tunnel/path.

A board-certified pathologist (J.M.) determined the depth of thermal effect histologically to be under 0.50 mm in all specimens (Table 1, Supplemental Figure 1) by measuring the distortion depth. The average maximal depth in subcutaneous tissue of thermal change for a 2-BEED was 0.009 mm at 20 W, 0.10 mm at 30 W, and 0.12 mm at 50 W power. The average maximal depth in subcutaneous tissue of thermal effect for a 3-BEED was 0.08 mm at 20 W, 0.07 mm at 30 W, and 0.10 mm at 50 W. A greater number of results were obtained at the 50 W power setting on the 3-BEED device because this was the highest electrosurgical power (worst-case combination scenario for regulatory testing of power setting and device) on which to test the BEED device; however, 50 W produced thermal effect readings that were less than the thickness of a first-degree, epidermal eyelid burn (thinnest human skin).

**Table 1.** Ex Vivo Thermal Damage Analysis

2-BEED				3-BEED			
Maximum thermal depth effect (mm)				Maximum thermal depth effect (mm)			
Reading no.	20 W	30 W	50 W	Reading no.	20 W	30 W	50 W
1	0.05	0.05	0.02	1	0.05	0.05	0.05
2	0.05	0.05	0.05	2	0.05	0.05	0.05
3	0.05	0.10	0.05	3	0.05	0.05	0.05
4	0.10	0.10	0.10	4	0.05	0.10	0.05
5	0.10	0.15	0.10	5	0.10	0.10	0.05
6	0.20	0.15	0.10	6	0.10	0.10	0.10
7			0.10	7	0.10	0.15	0.10
8			0.15	8	0.10	0.20	0.10
9			0.15	9	0.12	0.25	0.15
				10	0.15	0.30	0.15
				11	0.15	0.35	0.25
				12	0.22	0.35	0.30
				13			0.30
				14			0.35
				15			0.40
Average	0.09	0.10	0.09	Average	0.10	0.17	0.16
Median	0.08	0.10	0.10	Median	0.10	0.13	0.10
SD	0.06	0.04	0.04	SD	0.05	0.12	0.12

(A) Ex vivo thermal damage analysis: Maximum values and averages with SD. (B,C) Maximum thermal depth vs wattage in bar/symbol analysis for means with SDs. Maximum thermal depth vs wattage was not significantly different as per Kruskal–Wallis test. The values in bold summarize the contents of the table.

**Figure 3.**
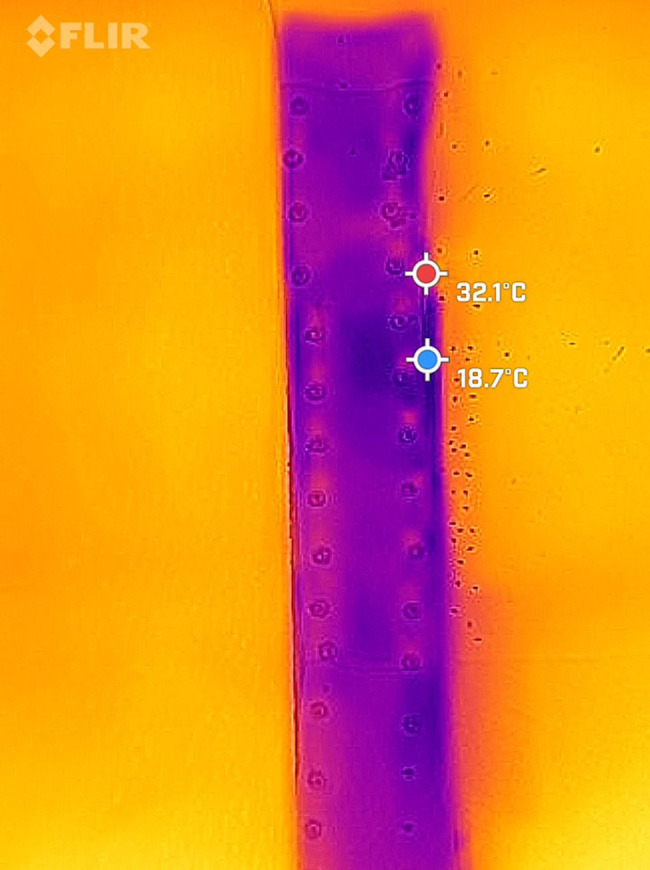
FLIR image of ex vivo abdominal wall with temperature minimum 18.7°C and maximum 32.1° C. FLIR, Forward Looking Infrared.

Figure 3 is a top FLIR thermal image of the fresh ex vivo abdomen model during 3-BEED model passage with a minimum tissue temperature reading of 18.7°C and a maximum reading of 32.1°C. In the ex vivo study (without tumescence) in which the wattages ranged from 20 to 50 W, no surface Forward Looking Infrared, FLIR readings increased more than 5°C above the starting temperature.

### In Vivo Porcine Model Results

Figure 5A and B (3 pages forward, for preoperative comparison with postoperative) is the top views of 2 posttattoo, immediately preoperative, porcine abdomens with 10 × 10 cm dot-tattooed grids. Figure 5E and F is immediately postoperative. Each pig had the subcutaneous tissue below 8 tattoo grids lysed with a 2- or 3-BEED device. The preponderance of ecchymosis occurred following spatula cannula instillation of tumescent anesthesia into the densely fibrous subcutaneous tissues in the abdomen on the left. In an attempt to mitigate bruising, a spinal needle was used to infuse the tumescent solution in Pig B; however, significant ecchymosis from tumescence still occurred. Some blanching of the skin following tumescence can be seen around Grid 5 and the contralateral grid on Pig B (Figure 5F, *) just from the epinephrine in the tumescent solution; however, this was seen to have been resolved upon inspection later on the day of the surgery.

Planar dissection was achieved using both distal (in forestroke) and proximal lysing segments (in retrograde stroke, with a force vector engaging the lysing segment). Complete liberation of the plane from fibrous attachments was verified by retrograde “clean-sweep” strokes, each with a greater portion of the force vector pointing laterally, toward the exposed proximal lysing segment.

**Figure 4.**
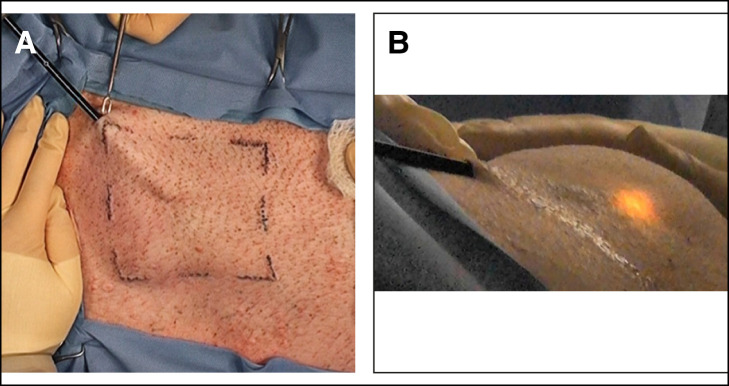
(A) 2-BEED intraoperative forward motion. (B) 2-BEED forward motion with operating room lights dimmed. Beaded Enhanced Electrosurgical Dissectors (BEED).

Figure 4A is a top intraoperative view of a 10 × 10 cm pig belly grid with a 2-BEED device in forward motion, the tip near the center of grid. Figure 4A also displays tenting of the skin and an assistant applying peripheral skin traction/tension. Traction toward the operating surgeon is facilitated by a double skin hook placed securely in the dermis of the entrance incision. The subject pig skin was much thicker and much more fibrous than average human abdominal skin. Despite the diminutive size, BEED tips were able to completely lyse numerous 100 cm^2^ areas in 0.8 to 3 min. Initial plane lysis passage was followed by a 1 min set of rearward “clean-sweep” strokes and to assure clearance of all fibrous tendrils within the boundaries of the tattoo grid. The BEED device passage speed ranged from 1 to 10 cm/s, depending on the resistance, power setting, and as confirmed by timed videography.

Figure 4B is a top perspective intraoperative view of a 10 × 10 cm pig belly grid with a 2-BEED device in forward motion. Operating room lights were dimmed to permit visualization and photography of electrosurgical discharge in the subcutaneous layer.

**Figure 5.**
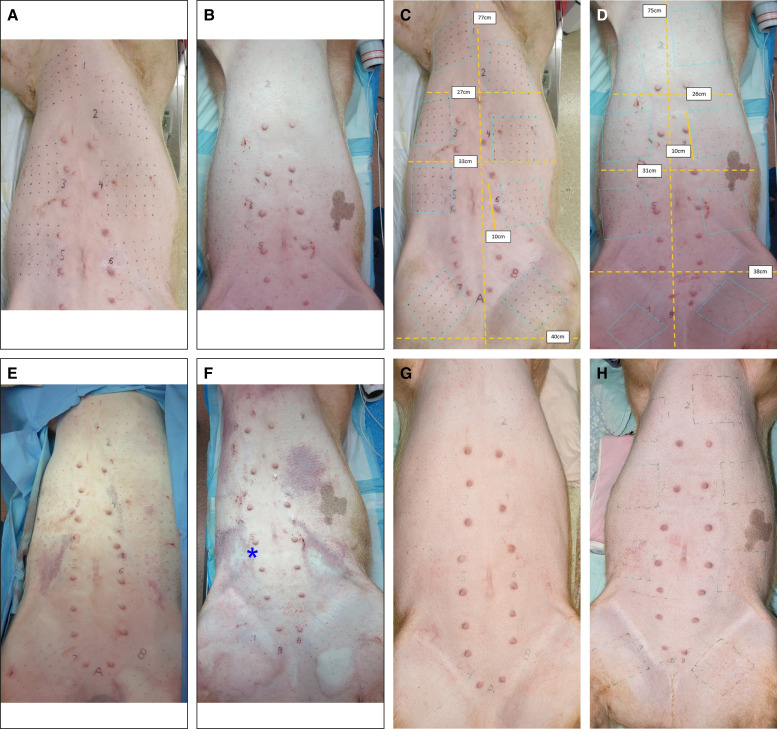
(A, B) Top views of porcine abdominal ventrums preoperative before tumescence; (C, D) preoperative before tumescence with superimposed neon-blue dotted parallelograms demonstrating total areas dissected as opposed to abdominal dimensions; (E, F) immediately postoperative; and (G, H) 90-days postoperative. Notice birthmark on left, mid-side of Pig B. The postoperative ecchymoses arose posttumescent infiltration and did not increase appreciably following BEED passage in the dense fibrotic tissue. Beaded Enhanced Electrosurgical Dissectors (BEED).

Figure 5C and D, of Pigs A and B, respectively, was taken preoperative before tumescence and has superimposed neon-blue dotted parallelograms, demonstrating total areas dissected as opposed to abdominal dimensions. Figure 5F and G shows immediate postoperative image.

### Flap Window In Vivo Grossing Immediately Postoperative

Videography was made of flap windows created over 10 × 10 cm live pig abdomen dissection grids immediately postoperatively demonstrating complete tissue plane separation and minimal lipolysis, char, or hemorrhage (see video link, 2m22s).

### Tissue Healing Results

Five to 8 mm width dissectors were passed at 1 to 2 cm/s in ex vivo models (1-10 cm/s in vivo models) with an average temperature rise of 5°C at 50 W. Immediately following in vivo dissections of 800 cm^2^ each (the majority of the ventral abdomen), subjects displayed preoperative behavior.

A total of 6 seromas developed between the 2 subject pigs, with 3 seromas on each pig. Three of the seromas formed at the grid sites located on the medial aspects of the thighs, and 3 on the grid sites located on the ventrums of the pigs. Five of the seromas resolved without intervention. Two seromas (1 medial thigh and 1 ventral) resolved by Day 14 postoperative, 2 ventral seromas resolved by Day 15 postoperative, and 1 medial thigh seroma resolved by Day 60. Ninety days ultrasound demonstrated zero seromas; however, histopathology discovered 2 flattened resolving, 2 cm seromas out of 16 dissection zones plus postpassage linear fibrosis.

The sixth seroma, located at a medial thigh grid, developed a dependent discolored area at approximately 20 days postoperative that progressed to a 2 cm, linear, dry necrotic. The pig had been seen to lay on this dependent traumatized seroma and periodically stepped on the seroma when standing up. The pig refused to accept compressive or protective dressings or medicated ointments. By 35 days postoperative, the necrotic patch separated from the surrounding skin and allowed draining of fluid from what was a seroma but then culture positive for *Streptococcus* Group C, *Staphylococcus aureus*, and *Pasteurella multocida*. Ceftiofur was administered empirically. Complete healing by secondary intention was achieved by 50 days postoperative. The maximum diameter of the necrosis was 2 cm which if circularized would estimate to be 3 cm^2^ of wound out of a total dissection size on the 2 pigs of 1600 cm^2^ which is approximately 0.00125% (or 1/8 of 1%). Hair growth was plentiful during the 3 months, indicating that BEED passage was not significantly detrimental to the hair follicles.

Figure 5G and H is the top view of the same 2 porcine abdomens 90 days postoperative. A total of 800 cm^2^ was dissected in each pig over a total of 20 min of instrument activation and passage time. The total abdominal and thigh ventral surface area on each pig was estimated to be 2100 and 2000 cm^2^. Thus, approximately 40% (1600/4100) of the pigs' soft ventrums were completely dissected (without Swiss-cheese tunneling). Subject pigs were eating and ambulatory within hours of surgery. The pigs increased 50% in weight over the 90 day period. No palpable nodules or abnormalities were noted. Following photography, B-mode, color Doppler, and power Doppler ultrasound images and cine loops of each grid were recorded. Similar ultrasound images and cine loops of control areas lateral and medial to the grids were also recorded. The pigs were euthanized, and samples were collected for histopathologic evaluation discussed below.

### Histopathology Results

**Figure 6.**
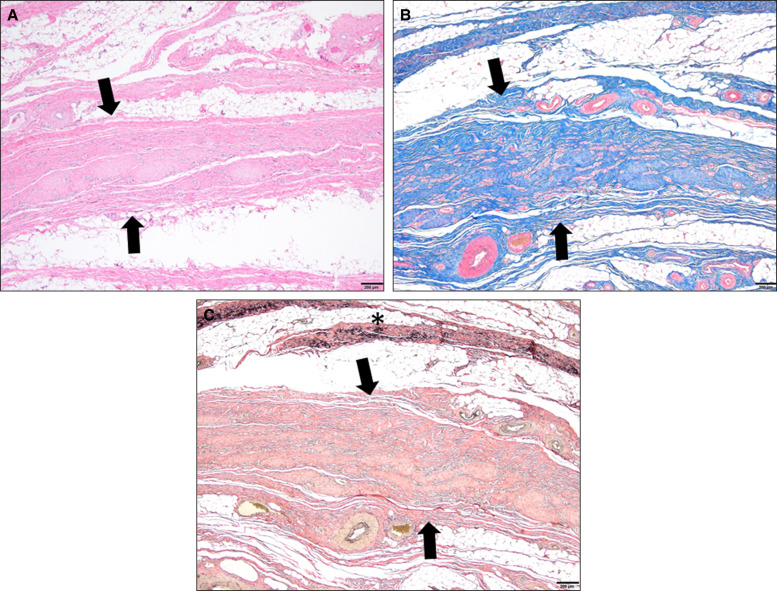
(A) Histologic evaluation of porcine 90 days postoperative sections demonstrated a linear band of fibrosis within the deep subcutaneous tissue (H&E, arrows). (B) Trichome stains collagen blue (arrows). (C) Elastin fibers were not present within the mature fibrosis (Verhoff Van Gieson, arrows); however, normal subcutaneous elastin fibers (black stain) were present within subcutaneous collagen bundles (*). Bar (bottom right) = 0.2 mm.

Figure 6A-C demonstrates the histopathologic effects using H&E, collagen, and elastin stains evaluated at 90 days postoperative from passage of a 2-BEED device at 50 W at average speed of 5 cm/s for 90 s in a 10 × 10 cm grid. In all skin samples, except for 1 (A4), fibrosis was deep to the dermis (within the subcutaneous, skeletal muscle, or deep to the skeletal muscle). In 2 samples (A1 and B2), resolving, collapsed, fluid-less subcutaneous seromas were identified. In total, the fibrosis was mature and there was a very little inflammation. There was no evidence of inflammatory fat necrosis, foreign body reaction, or significant inflammation in any section evaluated.

### Ultrasound Results

**Figure 7.**
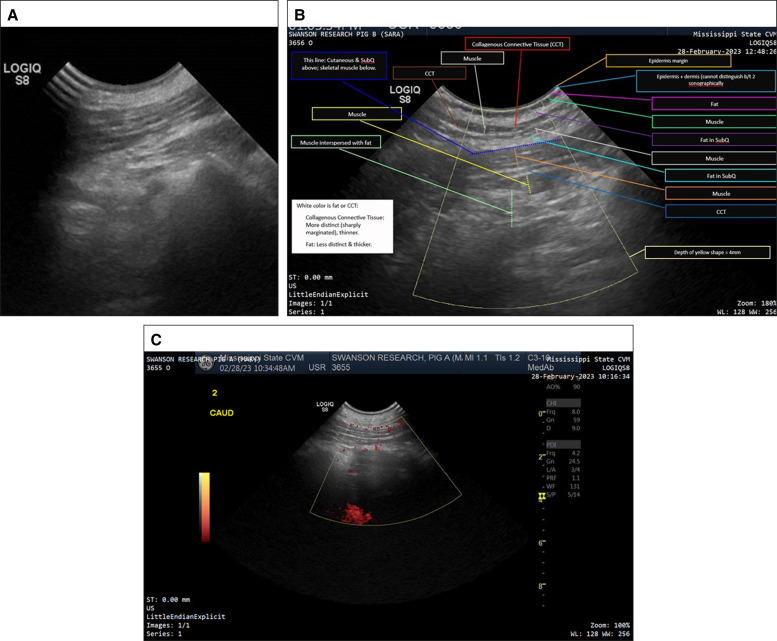
(A) Ninety days postoperative, diagnostic ultrasound image reveals no nodules, cysts, fluid collections, or tissue separations (as well as the remainder of the 16 dissected sites). (B) Labeled ultrasound layers display the normal, multilevel interpolation of numerous muscular bands throughout the subcutaneous fat. Note the labeling of the areas. (C) Ninety days postoperative in vivo Doppler ultrasound reveals slightly enhanced vascularity as per ultrasonographer and algorithm quantification.

Ultrasound images (Figure 7A-C) were taken 90 days postoperative demonstrating normal healing. No masses, fluid-filled cavities, or tissue separations were noted. Interrogation with color and power Doppler revealed normal blood flow in all areas.

### Forward Looking Infrared (FLIR) Results/Tissue Thermodynamics Results

In the in vivo study in which the wattages ranged from 20 to 50 W and gross tumescent anesthesia was used, no FLIR readings were seen above 37°C.

**Figure 8.**
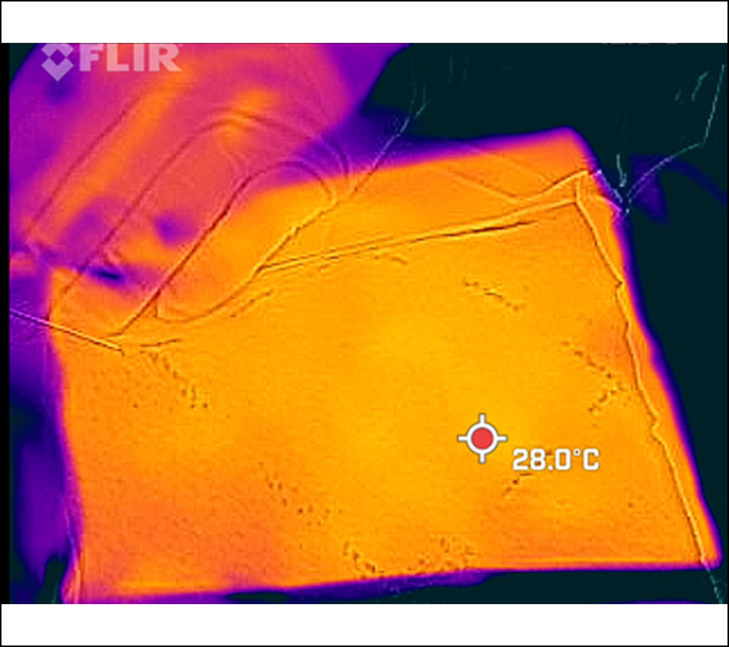
A top intraoperative view of a 10 × 10 cm pig abdomen area (boundary highlighted by Sharpie marker) undergoing subcutaneous dissection by a 2.5 BEED device. The highest temperature displayed in the grid was 28°C. Beaded Enhanced Electrosurgical Dissectors (BEED).

Figure 8 is a top intraoperative view (from a Teledyne-FLIR thermal camera; FLIR ONE Edge Pro) of a 10 × 10 cm pig belly area (boundary demarcated by Sharpie marker) undergoing subcutaneous dissection by a 2-BEED device. The highest temperature displayed in the grid was 28°C. Since the skin temperature of the periphery, which was not tumesced and often warmed by the assistant's hands, was nearly 38°C, on occasion the FLIR camera would register a temperature outside the grid as the highest, indicating the tumesced zone's temperature was lower. The FLIR ONE model produced a double image wherein the visible light input of its traditional lens is set askew to the infrared input of its thermal detector. As per FLIR: the FLIR ONE Pro packs-in 2 distinct cameras: one to capture infrared radiation, and another to capture visible light. Two cameras also allow the FLIR ONE Pro to perform the MSX image enhancement wherein the visible camera helps to locate edges of objects and then embosses this data into the thermal image in real time for a “real-world look.”

## DISCUSSION

The BEED electrosurgical dissectors were evaluated for subcutaneous plane dissections in porcine models. Additionally, a ballistic gelatin model with simulated collagen tendrils was used to document device activity and movement. In the fresh ex vivo porcine model, the extent of thermal effect was measured. Finally evaluated was an in vivo porcine model to demonstrate tissue thermodynamics, histology, and to elucidate surgical techniques.

A video file of the BEED dissector operation is available at BEED Dissectors: Pilot Studies.

**Figure 9.**
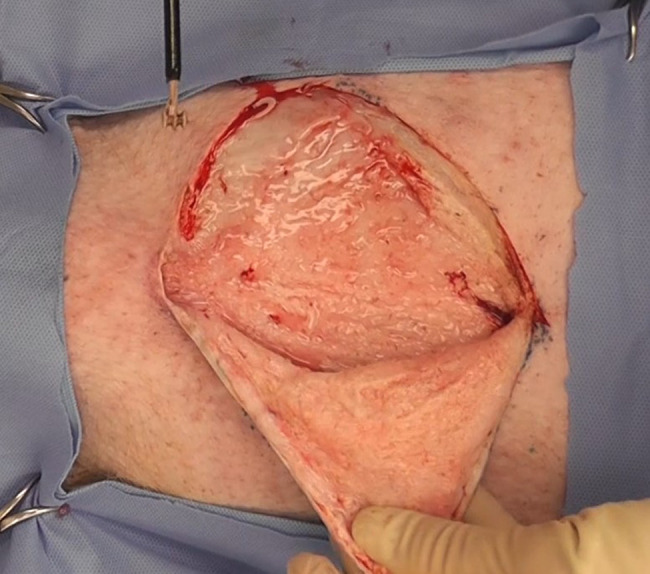
In vivo porcine abdomen 10 × 10 cm dissection plane (which was created with 3-BEED device [top left] in under 90 s) is opened (within 30 s of formation) by 2 side cutting and flap reflected to display minimal bleeding as well as completeness of dissection. Beaded Enhanced Electrosurgical Dissectors (BEED).

Based on these preliminary studies, it was determined that a pocket could be developed in the SAT with the BEED device that would cut/lyse the fibrous septae (which course vertically through the subcutaneous fat), without leaving “Swiss cheese-like” collagen fibrous remnants attached within the SAT. The completeness of a BEED dissection is demonstrated in Figure 9. Bleeding, blood staining, and hematoma were not encountered in the dissected areas. Bleeding was principally encountered at the edges in which a scalpel was used to create the observational flap.

The proposed technique with tumescent fluid infiltration may mitigate elevation of tissue temperatures with 100 mL epinephrine-containing tumescent infiltration per 10 × 10 cm grid. Appreciable electrosurgical smoke was not produced. No smoke evacuation devices were used.

Some have found PEAK PlasmaBlade dermal incisional damage on 6 W cutting current as 66 ± 5 μm and Valleylab pencil at 40 W in cut and coagulation modes, yielding 456 ± 35 μm and 615 ± 22 μm, respectively.^17^

Various devices, including an un-named electrocautery device, PEAK PlasmaBlade, Ultracision Harmonic Scalpel, and Argon Plasma Coagulation ranging from 40 to 80 W have been assessed for seromas, blood loss, hematomas, and drainage volume.^18^

Human, principally dermal incisions, made through an Erbe System displayed acute thermal injury of 686 ± 1037 μm at 80 to 180 W, and PEAK PlasmaBlade's resulted in 425 ± 175 μm thermal injury at 5 to 7 W.^19^

Smaller seromas were observed in human abdominal flap dissections through Colorado needle at power setting 35 (undisclosed generator) vs traditional scalpel.^20^

Other studies have evaluated human abdominoplasty wounds, showing higher rates of acute hemorrhage, lower limb thrombosis, pulmonary embolism, noninfectious/infectious collections, in addition to longer hospital stays, higher drain production, and increased postoperative visits in monopolar cutting mode electrosurgery as opposed to a No. 23 blade scalpel.^21^

A review, laser-assisted liposuction, cites the use of Cynosure's Smartlipo (max 18 W), Cynosure's Smartlipo-MPX (max 12 and 20 W at 1320 and 1064 nm wavelengths, respectively), CoolTouch's CoolLipo (max 15 W), and Osyrs/Med Surge Advance's Lipotherme (max 25 W) with listed complications.^22^

PlasmaJet vs monopolar dissections revealed significant dead space fluid collection wherein the mean total drained volume in PlasmaJet abdominal lipectomy was 322 mL vs 340 mL for monopolar lipectomy.^23^

At 90 days postoperative, subsurface ultrasonic imaging was useful to monitor healing after the BEED dissection procedure. Commonplace tissue findings of fluid, haziness from extracellular lipid, subcutaneous fat necrosis, or triglyceride cysts were not found. Ultrasonic Doppler studies demonstrated normal blood flow to the SAT in the treatment areas; thus, blood flow was not impaired according to Doppler ultrasound. This may be of significance regarding how the treated areas healed with limited collateral damage to adjacent SAT. The results were interpreted by the radiologist and compared pictorially.

Histologic sections of BEED dissections taken at necropsy (90 days posttreatment) demonstrated typical healing results without abnormal inflammation, scarring, and/or collagen deposition. One section demonstrated a small resolving seroma.

Six out of 16, 100 cm^2^ dissection zones developed seromas. Only one of the seromas was drained and the subject treated with an antibiotic. The subject was not anesthetized and moved substantially during the procedure and therefore no volumetric measurement of escaping fluid was possible. The 16 dissection zones were unprotected during the vast majority of after-care, because the pigs actively removed any dressings and potentially orally contaminated the areas. The pig cages had potential fecal and oral contamination in contact with all 16 zones. It is noteworthy that the sides of the cages were rods that could apply greater pressure to a wound than a flat surface, because the pounds per square inch would be higher along the edge of a rod. Thus, the 16 dissection wounds were subject to substantial trauma, pressure, and contamination risks. Fifteen out of 16 healed without incident and were undetectable ultrasonographically and barely detectable histologically at 90 days. The one wound that did develop necrosis developed typical necrotic findings only after the formation of a seroma that was seen to be stepped on by the pig more than once.

### BEED Tissue Thermodynamics

Tissue thermodynamic imaging with the FLIR did not detect skin temperatures higher than 42°C. The combination of limiting the duration of RF energy and keeping the BEED device in the plane of the SAT may prevent excessive skin temperatures that could produce a low temperature burn. This may be similar to what Ablaza et al.^24^ described with the use of tumescent fluid infiltration in ultrasonic-assisted liposuction. For BEED devices, use of tumescent fluid may permit volumetric energy redistribution, possibly decreasing target tissue density, cooling target tissue, and providing an ionic substrate for electrosurgical energy flow.

The effects of electrosurgical discharge into the 10 × 10 cm grids were studied with thermocouples (fresh ex vivo porcine models) and FLIR infrared camera imaging.

The following is a simple static computation of the amount of heat delivered into a fully tumesced tissue volume of dermis and subcutaneous tissue with an approximation of 1 cm^3^ of tumescent fluid being deposited underneath every 1 cm^2^ of surface area. Several assumptions were made. Please note that the device may be moved from 1 to 10 cm/s, with the higher end of the speed range lowering the temperature rise further.

Given: 1 cc = 1 g water; 4.18 J raises the temperature of 1 cc of water by 1°C; 1 J = 1 wW s.

Speed: The calculations below assume the device tip is moved at a constant speed of 1 cm/s and thus spends 1 motion-second beneath a 1 cm^2^ zone. However, the device tip is often moved, depending upon the quality of the target tissue, at speeds in excess of 2 cm/s. Therefore, the temperature rise will be halved just by moving 2 cm/s.

Given: a 3-BEED, 8 mm WIDE device tip is moved at 1 cm/s beneath a given square centimeter of surface area that has been tumesced (assumes 1.5× because of overlap).

Example 1: If moderate tumescence is 1 cm^3^ beneath 1 cm^2^ surface area, then 20 W applied (in 1 motion-second beneath 1 cm^2^ area) by the 3-BEED device is applied to recently applied room temperature moderate tumescence, then the expected increase in temperature is represented by: (20 J/4.18 J/cc) × 1 cc × 1.5) = 7.2°C. Assuming the start temperature is 20°C, then the final temperature would be 27°C.

Example 2: If turgor-tumescence is 2 cm^3^ beneath 1 cm^2^ surface area, then 40 W (in 1 motion-second beneath 1 cm^2^ area) applied by the 3-BEED device is applied to recently applied room temperature moderate tumescence, then the expected increase in temperature is represented by: (40 J/4.18 J/cc) × 0.5 cc × 1.5) = 7.2°C. Assuming the start temperature is 20°C, then the final temperature would be 27°C.

As per Table 1, the thermal effects resulting from passage of the BEED device in nontumesced ex vivo tissues at all 3 W was 0.12 mm which is approximately one-quarter of 0.5 mm. Statistical analysis, however, revealed no differences in thermal effect among 20, 30, and 50 W. This may perhaps be because of lower wattages reducing instrument passage speed and higher wattages increasing passage speed. Because BEED devices achieved relatively rapid dissection of pig skin, which is multiples denser than human tissue, it appears that wattages less than 20, 30, and 50 W may be found applicable for human usage.

Given the planar dissection capabilities of the BEED surgical platform, the authors plan on studying the platform in the following specialties:

- Plastic surgery: dissection of skin flaps; treatment of hyperhidrosis; breast and body contouring procedures (can be used open); debridement of necrotic tissue as a prep for closure-trauma/burns; breast and body contouring (to be determined. MJ—we do not have enough data to propose BEED application for nasolabial folds.)
- Interventional radiology/pain specialties: tunneling for device leads from generator to target organ/area.
- Neurosurgery spine: tunneling for cerebrospinal fluid drainage line to peritoneum; placement of power receiving antenna to charge implanted devices.
- Vascular surgery: tunneling for vascular grafts
- General surgery: tunneling for enteral feeding tube or peritoneal dialysis catheter.
- Cardiology: dissection of pacemaker pocket and automated external defibrillator pocket.

### Study Limitations

In the ex vivo study, no tumescence was used because on pilot testing, as almost all of the tumescent fluid immediately leaked from the subcutaneous edges of the specimen and was determined to be a concern for an electrical hazard to personnel. Unfortunately, in the ex vivo model, there appears to be no practical way to exactly mimic gross tumescence or to over-saturate in the fatty subcutaneous layer with significant water-based tumescent fluid diffusely, that would not accumulate as leakage. High-energy electrosurgical discharge disadvantageously affects most thermocouple read-outs in real time, and, therefore, the instantaneous (real-time) measurement of temperature is not practical. Therefore, a 1 to 3 s measurement delay in probe insertion was the most practical course.

The porcine model has long been appreciated by many scientists to be an excellent animal model for human skin. However, regarding surgical devices in the subcutaneous tissues, the porcine model displays densities of fibrous tissues and number of layers that are multiples of those found in typical human abdominal or extremity skin that has not been subject to trauma and resultant fibrosis. As per Figure 8B, over 9 different muscle and fibrous layers were ultrasonographically detected coursing the subcutaneous fat. The 16 in vivo porcine abdomen 100 cm^2^ dissections took between 0.8 and 3min to dissect followed by a 30 s checking dissection. Therefore, the passage of BEED devices in human subcutaneous tissue may be expected to encounter a fraction of the resistance experienced in the porcine models. As can be appreciated from the size of the BEED tips against the US dime coin in Figure 1A, the fact that the ceramic device tips can withstand 2 million pounds per square inch on nanoindentation studies was helpful in overcoming resistance without fracture or failure. Thus, BEED devices await human clinical studies to determine the speed at which various square centimeter dissections can be achieved.

Based on preliminary research in in vivo porcine tissues, it is believed that the BEED device and its variants give various surgical and interventional medical specialties the option of being able to rapidly create subcutaneous spaces in tissues with fewer limitations, such as tissue charring, desiccation, and hazardous surgical smoke from open use of electrosurgical devices as seen with older-generation cutting and energy-emitting instruments.

From his experience with beaded dissectors, co-author Schulman opines that BEED devices may mitigate “past-pointing” (at the far edges) in general surgery applications. BEED devices may also mitigate electrical discharge and attendant thermal effects throughout an entire subcutaneous dissection plane, especially those areas closest to the entrance incision in which the spoke-wheel pattern originates. The BEED devices may discharge most when fibers to be lysed are perpendicular to the tip plane and forced between the beads into contact with the lysing segments. Thus, if an area has already been dissected, it likely has no vertical attachments to contact lysing electrodes, thus less or no discernable discharge. Previously dissected areas are thus less likely to be energized multiple times, offering a degree of protection. Further studies to document this hypothesis may include low-light videography with mathematical assessment of light emission throughout the path of dissection. Also, robotically or mechanically affixed, remote-controlled, FLIR cameras may aid in more precise interoperative data capture.

Clinically, obvious seroma formation occurred in three-eighths of the wounds. In this study's surgical setting, 100% of the wounds were uncompressed, unprotected, subject to weight/trauma/shearing force, and in a dependent position (porcine abdomen) throughout over 99% of the postoperative period. At 90 days, no seromas were ultrasonographically detectable yet only discoverable microscopically.

Future studies anticipate determining whether the proximity to the entrance wound of spoke-wheel path overlap yields greater tissue thermal damage than more distant locations on the spoke-wheel pattern. This will be done on 10 × 10 cm dissections with 10 biopsies: 3 at 9 cm from the entrance wound, 3 at 5 cm from the entrance wound, 3 at 1.5 cm from the entrance wound, and a final biopsy 5 mm distal to the entrance wound. Five 10 × 10 cm dissections will be biopsied in the immediate postoperative period.

Regarding volumetric energy distribution and decreased tissue density because of tumescent fluids, this observation was made during the course of several years of prototype device testing in abattoirs with over 40 freshly deceased (usually under 30 min) pig specimens finding that those that were not tumesced required significantly greater force to operate the device for a given energy level. Less tissue density may imply that energy is less concentrated in a given area and thus distributed more expansively. Proving these concepts on a numerical basis is the object of future planned studies in which force gauges are integrated into the handle system and varying levels of tumescence are used in similar animals in similar body locations for a given set of wattages.

The ability of the long shaft (of which the distance to an entrance wound is adjustable by a snap handle) to bend or be directed within distant tissues from the entrance wound, for example, cellulite treatment, is anticipated to be tested using a series of vector-directed manual external pressures.

Assessment of time savings and rapidity of dissections in human subjects is anticipated for under 1 min axillary sweat dissection with varying degrees of tumescence and wattage levels.

Although the current sizing and geometry of the BEED tips that are controlled by CAD appear to be efficient at dissecting along and within some of the densest fibro-collagenous mammalian tissues and sequesters significant wattages from unwanted exposure to tissues, it is possible that further sizing and geometry changes may be optimized for various human subject ages, anatomical locations, previously operated fibrous sites, skin-density types, and previous disease. Thus, further studies with shape and size modifications may tailor-make tips for a variety of uses especially since the tips can be produced by 3D additive ceramic printing as well as traditional injection molding.

## CONCLUSIONS

BEED devices were studied in in vivo and ex vivo porcine abdominal subcutaneous models to access thermal injury and other aspects of tissue healing at 20, 30, and 50 W. Histologic studies in ex vivo porcine abdominal tissue revealed thermal damage measurements at or below those of other subdermal tissue treatments. Thermocouple studies of ex vivo tissues revealed temperature rises of 5°C on 50 W ESG blend settings. Histologic studies of in vivo tissues at 90 days revealed similar results. Ultrasonic studies at 90 days postoperative demonstrated normally healed tissues. Seromas were encountered that clinically resolved by 90 days as evidence by ultrasound. No bandaging or seroma mitigation techniques were used in this study. Further studies, in various closed and open surgical dissections, are anticipated in humans to determine safety, efficiency, and performance.

## Supplementary Material

ojae034_Supplementary_Data
