# Supplementary material for: Minimally Invasive Beaded Electrosurgical Dissectors, Basic Science, and Pilot Studies
Source: Aesthet Surg J Open Forum. 2024 May 6;6:ojae034. doi: 10.1093/asjof/ojae034 (PMC11210070; doi:10.1093/asjof/ojae034)
Supplement: ojae034_Supplementary_Data [file ojae034_Supplementary_Data.docx]

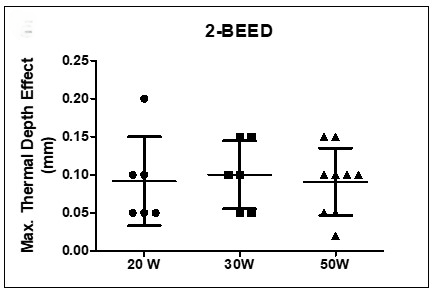

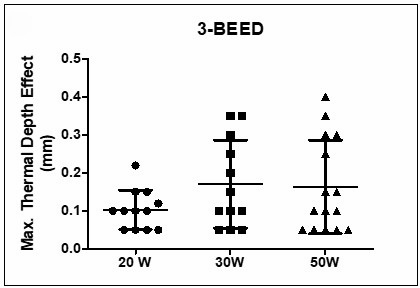


**B**

**A**

**Supplemental Figure 1.** Maximum thermal depth versus wattage in bar/symbol analysis for means with standard deviations. Maximum thermal depth versus wattage was not significantly different as per Kruskal-Wallis test.
